# Lycopene Is Enriched in Tomato Fruit by CRISPR/Cas9-Mediated Multiplex Genome Editing

**DOI:** 10.3389/fpls.2018.00559

**Published:** 2018-04-26

**Authors:** Xindi Li, Yanning Wang, Sha Chen, Huiqin Tian, Daqi Fu, Benzhong Zhu, Yunbo Luo, Hongliang Zhu

**Affiliations:** ^1^College of Food Science & Nutritional Engineering, China Agricultural University, Beijing, China; ^2^Key Laboratory of Beijing for Identification and Safety Evaluation of Chinese Medicine, Institute of Chinese Materia Medica, China Academy of Chinese Medical Sciences, Beijing, China

**Keywords:** lycopene, CRISPR/Cas9 system, genome editing, tomato fruits, carotenoid metabolic pathway

## Abstract

Numerous studies have been focusing on breeding tomato plants with enhanced lycopene accumulation, considering its positive effects of fruits on the visual and functional properties. In this study, we used a bidirectional strategy: promoting the biosynthesis of lycopene, while inhibiting the conversion from lycopene to β- and α-carotene. The accumulation of lycopene was promoted by knocking down some genes associated with the carotenoid metabolic pathway. Finally, five genes were selected to be edited in genome by CRISPR/Cas9 system using *Agrobacterium tumefaciens*-mediated transformation. Our findings indicated that CRISPR/Cas9 is a site-specific genome editing technology that allows highly efficient target mutagenesis in multiple genes of interest. Surprisingly, the lycopene content in tomato fruit subjected to genome editing was successfully increased to about 5.1-fold. The homozygous mutations were stably transmitted to subsequent generations. Taken together, our results suggest that CRISPR/Cas9 system can be used for significantly improving lycopene content in tomato fruit with advantages such as high efficiency, rare off-target mutations, and stable heredity.

## Introduction

Precise genome editing provides remarkable advantages in plant agricultural trait improvement by generating tailored modifications at a target sequence. Clustered regularly interspaced palindromic repeats/associated 9 (CRISPR/Cas9) has been developed from studies on the prokaryote-specific adaptive immune system, where endonuclease Cas9 is coupled with a synthetic single guide RNA (sgRNA), generating an RNA-guide nuclease. The specificity of Cas9-directed DNA double-strand cleavage, causing double-strand breaks (DSBs) in nuclear DNA, is defined by Watson–Crick base-pairing of a 20-bp guide sequence on the guide RNA (gRNA) and a PAM, (“NGG” motif) immediately downstream of the target region. DSBs induced by CRISPR/Cas9 can trigger two independent endogenous DNA repair pathways: NHEJ and HR. NHEJ is error-prone and frequently causes indels around the DSBs, whereas HR accurately repairs DSBs by using the homologous flanking sequence or an exogenous donor DNA as a template, which can frequently result in small or large chromosomal changes (Sonoda et al., 2006; Wyman and Kanaar, 2006; Gaj et al., 2013; Voytas, 2013). CRISPR/Cas9 genome site-specific editing technology, a new genome modification tool, has been successfully applied to various plants, including rice (Miao et al., 2013), wheat (Shan et al., 2013; Wang et al., 2014), maize (Liang et al., 2014), and tomato (Brooks et al., 2014; Cermak et al., 2015; Ma et al., 2015; Ito et al., 2015; Jacobs and Martin, 2016; Lin et al., 2016; Xu et al., 2016). Unlike previous genome editing tools such as ZFN and TALEN, the CRISPR/Cas9 is easier to use, design flexible, and very cost-effective. Moreover, CRISPR/Cas9 genome editing tool is characterized by high mutation efficiency as well as rare off-target mutations (Xu et al., 2015), and homozygous mutations at the desired sites can be transferred to the subsequent generation owing to stable inheritance (Zhang et al., 2014; Fan et al., 2015). Furthermore, transgene-negative plants were observed in nearly all low-copy T_1_ generation of rice after CRISPR/Cas9-mediated targeted mutagenesis (Xu et al., 2015), which promoted the application of CRISPR/Cas9. CRISPR/Cas9 is also widely used in tomatoes. The mutations mediated by CRISPR/Cas9 are diverse; Rodriguez-Leal et al. (2017) reported that CRISPR/Cas9 can carry multiple gRNAs to rapidly and efficiently generate dozens of alleles for genes in order to interpret their function better. In addition, functional knockout mediated by CRISPR/Cas9 is significant. Ito et al. (2015) reported the CRISPR/Cas9 system can efficiently induce mutations in the tomato *RIN* gene and affected the accumulation or structure of the RIN protein. Yang et al. (2017) showed that maturation was significantly inhibited in mutants after RNA editing factor SlORRM4 was knocked out by CRISPR/Cas9 in tomato. However, CRISPR/Cas9 genome editing also has certain limitations, the genotypes cannot be artificially controlled, and production of large numbers of homozygotes often requires propagation over several generations.

**FIGURE 1:**
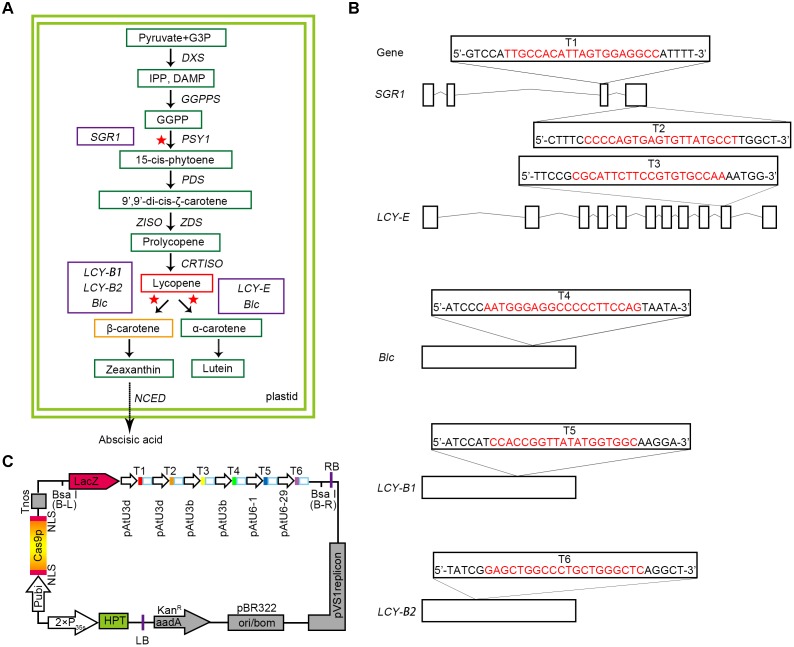
Selection of target genes and designing of CRISPR/Cas9 binary expression cassette. **(A)** A map of the target genes in the carotenoid metabolic pathway. The green boxes represent the key substances in the metabolic pathway. The red and orange boxes show the two substances, lycopene and β-carotene, respectively. A solid arrow indicates a direct effect, and a dashed arrow indicates an indirect effect. The selected target genes are represented by purple boxes, and the red asterisks represent the sites at which the target genes act on the pathway. G3P, glyceraldehyde 3-phosphate; DXS, 1-deoxy-D-xylulose 5-phosphate synthase; GGPPS, geranylgeranyl pyrophosphate synthase; PDS, phytoene desaturase; ZISO, z-carotene isomerase. **(B)** Five target genes were selected according to the synthesis and metabolism pathways of lycopene, and six target sites were designed. The target sequences are marked in red, and small rectangle frames indicate the PAM. Straight lines and boxes are the introns and exons of the target genes, respectively. **(C)** Structures of the pYLCRISPR/Cas9-Lycopene binary vectors. HPT(-H) encodes hygromycin B phosphotransferase. The six targets designed are represented by solid boxes in different colors, and the promoters used for each target are shown.

Lycopene is considered as a bioactive component for treating chronic diseases and lowering the risk of cancer and cardiovascular diseases (Li and Xu, 2014; Pouchieu et al., 2014; Tang et al., 2014); numerous studies have attempted to elucidate the pathways associated with lycopene metabolism. It is a C_40_ carotenoid and is synthesized via carotenoid metabolism during fleshy fruit ripening (**Figure 1A**). Carotenoid biosynthesis depends on isopentenyl diphosphate (IPP) and its isomer DMAPP (Chappell et al., 1995). In plastids, four molecules of IPP were condensed to a molecule of GGPP. Then, two molecules of GGPP can be catalyzed by phytoene synthase 1 (PSY1) to form a molecule of colorless 15-*cis*-phytoene, which are head-to-head condensed, leading to the generation of ζ-carotene and pink prolycopene. Prolycopene is then converted to all-*trans*-lycopene by carotenoid isomerase (CRTISO). Next, LCY-B and LCY-E catalyze the cyclisation of lycopene to produce β-carotene or α-carotene, respectively. Eventually, these substances can be degraded into lutein, zeaxanthin, and other carotenoids. Lycopene, a major component of carotenoids in ripe tomato, confers the attractive color and economical quality to the fruit. Thus, enhanced lycopene accumulation in fruit is essential to improve the visual and functional properties of tomato.

Modulation of the expression of key genes in the lycopene metabolism pathway is an effective way to increase lycopene content. Recently, significant progress has been achieved in genome modification in plants (Giuliano et al., 1993; Fraser et al., 1994, 1999; Rosati et al., 2000; Galpaz et al., 2008; Chen et al., 2015). For example, null mutation of the gene lycopene β-cyclase 2 (*LCY-B2*) in transgenic plants increased the lycopene content by about 5% on average and led to the formation of deep-red colored tomato fruits (Ronen et al., 2000). Further, the overexpression of phytoene synthase 1 gene (*PSY1*) significantly increased the level of lycopene in tomato fruit (Fraser et al., 2007). In addition, fruit-specific RNAi-mediated suppression of 9-*cis*-epoxycarotenoid dioxygenase 1 (*NCED1*) produced deep-red colored fruits with high accumulation of lycopene and β-carotene (Sun et al., 2012). Moreover, silencing of stay-green 1 (*SGR1*) promoted the activity of PSY1 via direct physiological interaction during fruit maturation, which significantly elevated the accumulation of lycopene and β-carotene (four and ninefold, respectively) in fruit (Luo et al., 2013). However, recent studies on enhancing lycopene accumulation in tomato fruit by regulating the carotenoid metabolic pathway are mainly focused on the modification of a single related gene. Modulation of multiple genes in metabolic networks to achieve the accumulation of lycopene has been rarely reported, owing to the complexity and integrity of the metabolic mechanism. Thus, in this study, CRISPR/Cas9 was applied to regulate multiple genes associated with the carotenoid metabolic pathway of tomato, in order to increase the lycopene content in fruits and explore the roles of specific key genes in carotenoid metabolism. On the one hand, we lay the foundation of research for the application with CRISPR/Cas9 multiplex genome editing in metabolic pathway. On the other hand, we provide a new research idea for engineering the content of some target substance in the metabolic pathway.

## Materials and Methods

### Plant Materials and Growth Conditions

The study was conducted using *Solanum lycopersicum* cv. AC. All plants were grown in the same greenhouse (greenhouse conditions: 16 h light/8 h dark, 25°C). Tissue samples at 7 days after breaker stage of ripening (Br+7) of fruits were collected and immediately frozen in liquid nitrogen and stored at -80°C. Mature seeds were collected from T_0_ plants, dried, and shaken in a 25°C shaker. The germinated seeds were then planted in the soil, and seedlings were grown under the above-mentioned culture conditions.

### Selection of sgRNA Target Sequence and pYLCRISPR/Cas9-Lycopene Vector Construction

CRISPR-P^1^ was used to select specific sgRNAs targeting *SGR1* (GenBank accession no. DQ100158), lycopene ε-cyclase (*LCY-E*; GenBank accession no., EU533951), beta-lycopene cyclase (*Blc*; GenBank accession no., XM_010313794), lycopene β-cyclase 1 (*LCY-B1*; GenBank accession no., EF650013), and *LCY-B2* (GenBank accession no., AF254793) (Supplementary Table S1). Improper GC content has been shown to lead to inefficient editing (Wang et al., 2014); therefore, the content of GC was between 55% and 75%. In addition, 4 or more consecutive T nucleotides in the target sequence were avoided since they would be recognized as transcriptional termination signal by RNA polymerase III. Furthermore, RNA Folding program^2^ was used to ensure that no more than five base pairings occurred between the target sequence and sgRNA sequence since the secondary structure of sgRNA was remarkably affected by the editing efficiency (Makarova et al., 2011). In order to obtain a more obvious effect on gene function, we selected target sites in the exon of the target genes. Finally, targets with fewer putative off-target loci were preferred.

The pYLCRISPR/Cas9-Lycopene vector was constructed as described previously (Ma et al., 2015). First, each target sequence was ligated to its corresponding sgRNA expression cassette during the first PCR. This was followed by a second PCR to amplify the fragments as well as induce BsaI restriction sites in the target for generating of sgRNA expression cassettes with target sequences. The standard PCR condition was as follows: 94°C for 2 min; 94°C for 10 s; 55°C for 30 s; 68°C for 20 s for 28 cycles and 72°C for 7 min. Finally, the sgRNA expression cassettes were assembled to pYLCRISPR/Cas9 binary plasmid in one round of cloning by using Golden Gate ligation for six sgRNAs expression cassettes in pYLCRISPR/Cas9-Lycopene vector. The reactions were incubated for three cycles (37°C, 10 min; 10°C, 5 min; 20°C, 5 min) and a subsequent 10 cycles (37°C, 3 min; 10°C, 5 min; 20°C, 5 min) was run. Then the reaction was incubated at 37°C, 5 min. The oligonucleotide primers used are listed in Supplementary Table S2.

### Plant Transformation

The *Agrobacterium*-mediated transformation method (Van Eck et al., 2006) was used, and pYLCRISPR/Cas9-Lycopene plasmid was transformed into AC. In brief, tomato seeds were germinated on MS medium after sterilization with 4% NaClO. After 7–10 days culture, the apical segments of hypocotyls were punctured with OD_600_ = 0.5–0.6 of *Agrobacterium* suspension. Then, the explants were inoculated on selective plates with hygromycin (10 μg/mL) until transgenic plants were regenerated from the calluses. After *in vitro* regeneration, plants were transplanted into soil in light growth chamber.

### DNA Extraction and Mutation Detection

About 1–5 mg fresh frozen leaves were used for DNA extraction by using hi-DNA secure plant kit (Tiangen, Beijing, China). The extracted genomic DNA was then used as a template to amplify the relevant fragments from each of the target genes by using primers (Supplementary Table S3) flanking the target sites. The standard PCR condition was as follows: 94°C for 3 min; 94°C for 30 s; 55°C for 30 s; 72°C for 30 s for 35 cycles and 72°C for 7 min. The PCR products were sequenced directly by using internal sequencing primers (Supplementary Table S3) or cloned into the pEasy-T1 (TransGen Biotech, China) vector and then sequenced using the Sanger method to identify mutations. Superimposed sequence chromatograms produced by biallelic and heterozygous mutations were decoded using DSDecode^3^ and manual analysis. The mutation rate of each target is calculated by the ratio, which is the number of transgene tomato plants edited at each target to 24 (the number of total transgenic tomato plants obtained).

### *Cas9* and Off-Target Analysis

In all, 10 mutants were selected randomly from lycopene mutants used for the detection of *Cas9* gene by PCR. The standard PCR condition was as follows: 94°C for 3 min; 94°C for 30 s; 55°C for 30 s; 72°C for 30 s for 35 cycles, and 72°C for 7 min. Primers used for amplification of *Cas9* gene are listed in Supplementary Table S6.

The lines were selected from the plants in which the targets were edited, based on the first two candidate off-target sites predicted by the online tool (CRISPR-P^4^), to sequence the changes in the fragment amplified by PCR from its corresponding gene locus. The standard PCR condition was the same as *Cas9* analysis. Primers used for off-target site mutation analysis are listed in Supplementary Table S4.

### Carotenoid Extraction and RT-HPLC Analysis

The carotenoid was determined by grounding the samples into powder after freezing in liquid nitrogen. About 0.2 to 0.5 g tissue powder was dissolved in 1 mL of acetone and centrifuged. The tissue debris was dissolved again in 1 mL of dichloromethane and centrifuged. The supernatant was pooled using an acetone filtrate. The dissolving and collection of solvents was repeated until the tissue lost its color. Pigments were extracted by partitioning the solvent mixture against an equal volume of diethyl ether and 0.2 volume of 12% w/v NaCl/H_2_O. The colored organic fraction (upper phase) was collected and dried under a stream of N_2_ and the dry lipid extract was re-dissolved in 1 mL acetone for further analysis.

Carotenoid were analyzed using reverse-phase high-performance liquid chromatography (RT-HPLC) as described in a previous study with some modifications (Neuman et al., 2014). Chromatography was performed using an Agilent liquid chromatography system (Agilent Technologies, Santa Clara, CA, United States) equipped with a model G1322A degasser, model G1311A infusion pump, model G1313A auto sampler, model G1316A column thermoformer, and model G1314A detector.

The static phase consisted of a Diamonsil C18(2) reversed-phase column (4.6 mm × 250 mm, 5 μm; Dikma). The mobile phase consisted of eluent A (acetonitrile: H_2_O; v/v; 9:1) and eluent B (ethyl acetate) at a constant flow of 1 mL⋅min^-1^. The linear gradient program was performed as follows: initial condition was 55% A to 100% B within 30 min and back to the initial condition for re-equilibration. Analysis was conducted under subdued light to avoid carotenoid degradation.

HPLC-grade β-carotene and lycopene standards were obtained from Sigma (St. Louis, MO, United States).

### Carotenoid Extraction and HPLC-MS Analysis

Carotenoid extractions were performed as described previously (Fantini et al., 2013). Briefly, lyophilised tomato fruit powder was extracted with chloroform and methanol (2:1 by volume). Subsequently, 1 volume of 50 mM Tris buffer (pH 7.5, containing 1 M NaCl) was added, and the samples were kept for 20 min on ice. After centrifugation (15 000 *g* for 10 min at 4°C), the organic phase was collected and re-extracted. The combined organic phases for each sample were then dried by nitrogen blowing and re-suspended in 100 μL of ethyl acetate. For each group, at least three independent extractions were performed. The carotenoids were identified and quantified using an Accurate-Mass HPLC1200/MS-QTOF 6520A (Agilent Technologies, United States) system packed with a reversed-phase column (4.6^∗^150 mm, 3 μm; YMC, Japan), and the carotenoid was washed out with the mobile phase: *A* = 81% MeOH + 15% MTBE + 4% H_2_O; *B* = 8% MeOH + 90% MTBE + 2% H_2_O at a flow rate 0.4 mL/min. The settings were as follows: DAD, 260–550 nm; mass range, 200–800; APCI ion source, drying gas of N_2_ at the pressure of 40 psi, 350°C, 8 L/min; VCAP, 3500 V; Fragmentor, 160 V; Skimmer, 65 V; and OCT RF Vpp, 750 V, with the negative MS scan mode 2GHzExt Dyn (3200). HPLC peak areas at 260–550 nm was integrated and calibrated using external standards (e.g., α-carotene purchased from Sigma-Aldrich). The calibrated samples were mixed to generate multiple-diluted external calibration curves for the quantification of the pigments. Carotenoids were identified based on typical retention times and specific published absorption spectra (Mialoundama et al., 2010; Regnier et al., 2015).

### Transmission Electron Microscope Analysis

Transmission electron microscopy (TEM) results were analyzed as described in a previous study (Yang et al., 2017); the pericarps of fruits from WT and mutants at Br+7 were fixed with 2.5% glutaraldehyde for over 2 h and washed three times with 0.1 mL phosphate buffer. Next, the sample was fixed with 1% osmic acid for 2 h and washed three times with 0.1 mL phosphate buffer. The sample was dehydrated using different concentrations of acetone (30, 50, 70, 90, and 100%). Next, it was embedded and aggregated with ethoxy line resin and sliced up using LEICA UC6 ultra microtome. After the sample was dyed with both uranyl acetate and lead citrate, the prepared sample was observed under a JEM-1230 transmission electron microscope.

## Results

### Selection of Key Target Genes in the Synthesis and Metabolism Pathways of Lycopene

Based on the advantages of CRISPR/Cas9 multiplex genome editing system, we carefully selected a bidirectional strategy (**Figure 1A**). Some sites were designed to target gene *SGR1* for promoting the synthesis of lycopene, whereas others were selected from genes that catalyze the cyclisation of lycopene, such as *LCY-E*, *LCY-B1*, and *LCY-B2*, as well as *Blc. LCY-E* was used to prevent the cyclisation from lycopene to α-carotene and *LCY-B1* and *LCY-B2* were used to prevent the cyclisation from lycopene to β-carotene (Pecker et al., 1996; Ronen et al., 1999, 2000; Moreno et al., 2013; Ralley et al., 2016), whereas *Blc* is a gene that has both β-cyclase and ε-cyclase activities predicted by InterPro Database^5^.

Since *SGR1* gene plays a relatively important role in the accumulation of lycopene, two target sites (targets T1 and T2) were designed to ameliorate the targeting efficiency of this gene (**Figures 1A,B**). In addition, the other corresponding target sites were selected (targets T3, T4, T5, and T6) at the exons of genes *LCY-E*, *Blc*, *LCY-B1*, and *LCY-B2*, respectively (**Figures 1A,B**). Finally, 6-sgRNA of the CRISPR/Cas9 binary expression system (**Figure 1C** and Supplementary Table S1) was constructed to target distinct tomato genomic sites of genes associated with the synthesis and metabolic pathways of lycopene, including both multiple genes in a pathway and multiple sites in a single gene.

### Efficient Multiplex CRISPR/Cas9-Mediated Targeted Mutagenesis in T_0_ Tomato Plants

**FIGURE 2:**
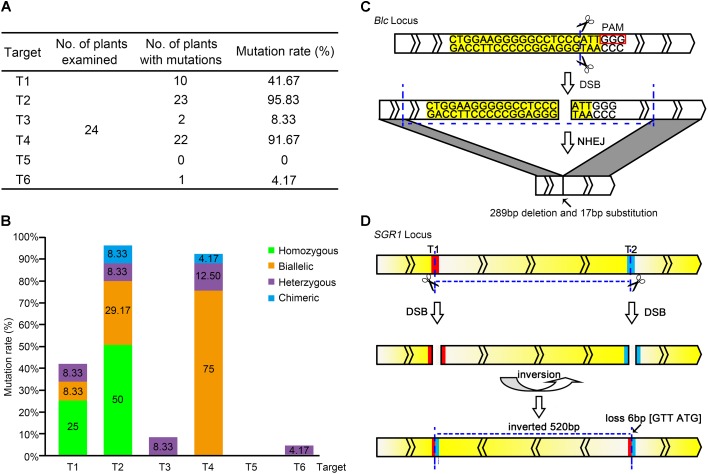
Editing of each target site in the pYLCRISPR/Cas9-lycopene expression cassette. **(A)** Total editing efficiency of the six targets in the pYLCRISPR/Cas9-lycopene expression cassette. The mutation rate is the ratio of the number of mutations detected to that of the total number of plants in which mutations are detected. **(B)** Specific types of each target among the six target sites in the pYLCRISPR/Cas9-lycopene expression cassette. Green, orange, purple, and blue represent homozygous, biallelic, heterozygous, and chimeric mutations, respectively. **(C)** Large fragment deletion on *Blc*. Target sequence is labeled with yellow. Small rectangle frames indicate the protospacer adjacent motifs. The transcriptional direction of the *Blc* is shown. **(D)** Special DNA inversion on *SGR1*. The red and blue parts represent the targets T1 and T2, respectively. Gradient change of color between yellow and white indicates the transcriptional direction of *SGR1*.

The editing efficiency of CRISPR/Cas9 for the selected target genes has been shown to be remarkable (Brooks et al., 2014; Zhang et al., 2014; Fan et al., 2015). In all, 24 lines of T_0_ transgenic plants (CR-lycopene-1 to 24) were obtained; specific editing types of each target were identified and analyzed. Fortunately, the 24 transgenic lines showed different types of genome editing at the target sites (Supplementary Figure S1), which indicated that multiplex CRISPR/Cas9 is extremely efficient in tomato fruit to generate tailor-made modifications at target sequences. The mutation rates varied widely among different target sites, from 0 to 95.83% (**Figure 2A**). The editing efficiencies of targets T2 and T4, especially target T2 (95.83%), were considerably higher than those of the others. However, target T5 did not show gene editing in any of the obtained transgenic lines (**Figure 2A**).

Further analysis of the RNA-guided genome-editing events indicated that the editing type also varied among distinct targets (**Figure 2B**). Two kinds of mutations, homozygous and biallelic mutations, were the most common among six target sites. Generating homozygous mutations at targets T1 and T2 with higher editing efficiency was possible. However, target T4 was slightly distinct, and the biallelic mutation was dominant. Correspondingly, the mutation type of targets T3 and T6 with low editing efficiency was not ideal; it was dominated by heterozygous mutations. In our study, targets T1 and T2 driven by the AtU3b promoter and target T4 driven by the AtU3d promoter showed high editing efficiencies. In contrast, target T5 regulated by the AtU6-1 promoter and target T6 regulated by the AtU6-29 promoter showed very low editing effects.

Although most gene mutant alleles were small indels (less than 10 bp) at the desired target sites, two interesting mutation types were observed. One was a large DNA fragment deletion. The length of PCR fragments in target T4 was obviously shorter, suggesting the deletion of a large DNA fragment with different sizes on target gene (**Figure 2C** and Supplementary Figure S2). Another was DNA inversion (**Figure 2D**), where the direct and complementary strand exchange occurred between targets T1 and T2 of *SGR1* gene.

In addition, we found that the percentage of T_0_ plants carrying mutations at two target sites was similar to the expected double mutation rate (Supplementary Table S5), indicating that mutations at two sites targeted by one construct might occur independently of each other. This finding is similar to those reported previously (Zhang et al., 2014). This result suggests that the levels of Cas9 and sgRNAs were not limited in transgenic tomato plants.

### The Detection of *Cas9* Gene and Off-Target Analysis in CRISPR/Cas9-Mediated Lycopene Mutants

The presence of Cas9 in lycopene mutants was confirmed by PCR for *Cas9* gene in the randomly selected lycopene mutation lines (primers listed in Supplementary Table S6). The *Cas9* gene was detected in the lycopene mutants (Supplementary Figure S3), indicating that the CRISPR/Cas9 binary expression cassette was indeed delivered to the tomato plant cells by *Agrobacterium tumefaciens*.

The potential off-target effects of CRISPR/Cas9 in tomato were evaluated by detecting the editing of putative off-target sites (Supplementary Table S7). No clear off-target events were detected, suggesting that CRISPR/Cas9-induced mutagenesis was highly specific in tomato plants. Previous studies have also shown similar results that CRISPR/Cas9 system has obvious advantages of avoiding off-target mutations in tomato (Zhang et al., 2014). The CRISPR/Cas9 multi-target gene editing technology with well-designed specific sgRNAs has distinct characteristics of significant editing and low off-target mutations.

### Lycopene and β-Carotene Contents of Mutant Fruits Were Remarkably Enhanced by CRISPR/Cas9-Mediated Gene Editing

**FIGURE 3:**
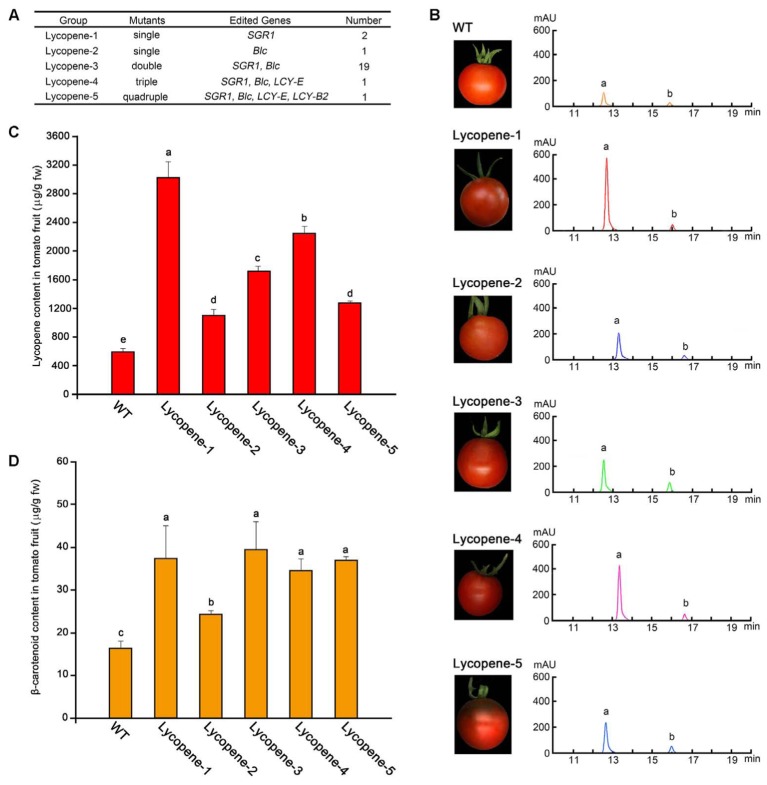
Determination of lycopene content in tomato fruit at Br+7 from different mutant groups. **(A)** List of different groups of lycopene mutants according to the different combinations of mutant genes. **(B)** HPLC results of crude extracts from tomato fruit samples in different groups. a, b, lycopene and β-carotenoids, respectively. **(C,D)** Contents of lycopene and β-carotenoids of tomato fruit in the five different groups and WT, respectively. Error bars represent standard deviation. Different lowercase letters show statistically significant difference according to ANOVA followed by Duncan’s test (*p* < 0.05).

The levels of carotenoids were determined by classifying the 24 transgenic tomato plants into 5 mutant groups according to the different mutant target genes, including single, double, triple, and quadruple mutants, which were named as Lycopene-1 to 5 (**Figure 3A**). Representative transgenic lines were selected from each group for further analysis. The tomato fruits of different mutant groups at Br+7 of ripening were sampled separately for determining lycopene and β-carotene contents by using HPLC. The levels of two main components of the tomato fruit were determined according to the respective peak of the commercial standard of lycopene and β-carotene (**Figure 3B**). HPLC analysis showed that the contents of lycopene and β-carotene in all lycopene mutants were higher than those in the WT plants (**Figures 3C,D**). In particular, the lycopene content of group Lycopene-1, in which the *SGR1* gene was targeted alone, was the highest. Correspondingly, the color of Lycopene-1 fruit was also the most vivid (**Figure 3B**), and the lycopene level (5.1-fold) in Lycopene-1 mutants was slightly higher than that (4-fold) in a previous transgenic tomato fruit subjected to RNAi of *SGR1* (Luo et al., 2013). However, mutation of the *Blc* gene alone (Lycopene-2) did not remarkably improve lycopene accumulation compared with that in the Lycopene-1 group (**Figure 3C**), indicating that the effect of *SGR1* gene on the regulation of lycopene content in tomato fruit was more pronounced than that of *Blc* gene.

The content of other carotenoids in the metabolic pathway was determined using HPLC-MS (Supplementary Figure S4). The results showed that the content of phytoene, prolycopene, α-carotene, and lutein in most of the lycopene mutants enriched significantly compared with in WT. Even the contents of these carotenoids in the single mutants also increased significantly, such as Lycopene-1 and Lycopene-2. However, the Lycopene-4 mutant showed a significant decrease in α-carotene content compared to that in WT. Moreover, unlike in other mutants, prolycopene in Lycopene-4 mutant showed no significant change from that in WT.

**FIGURE 4:**
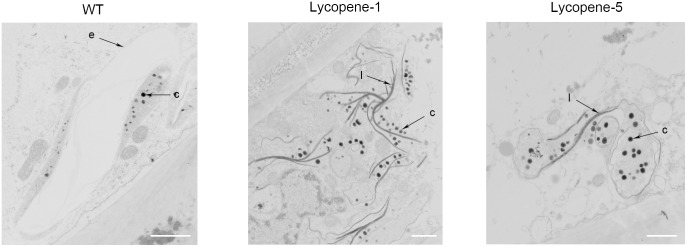
TEM images of epidermal cells in tomato fruit of WT and lycopene mutants at Br+7. c, carotenoid containing structures; e, plastid envelope; l, crystal line. Bar, 1 μm.

To further confirm the improvement of lycopene accumulation at the cellular level, we used TEM to observe the tomato fruits of WT, Lycopene-1, and Lycopene-5 (**Figure 4**). Unlike in WT, in Lycopene-1 and Lycopene-5, the plastid numbers in the pericarp cells were significantly higher at Br+7. Further, carotenoid-containing structures (osmiophilic globules) and crystal lines were higher in the plastids of lycopene mutant fruits than in WT fruits (**Figure 4**), resulting in the higher lycopene content of mutant tomatoes. In addition, the TEM images were different between the mutant and WT fruits at Br+7 (**Figure 4**). At the ripening stage, the vacuolisation of chloroplast was evident in the WT fruits, but a complete membrane was still observed. However, only plastids, but not chloroplast, were observed in the mutants. Our findings indicated that, with increased lycopene concentration in fruits, the conversion of chloroplasts to chromoplasts might occur earlier in transgenic fruits than in WT. This is sufficient to show that CRISPR/Cas9 multi-target genome editing can be successfully applied to regulating the metabolism of carotenoids to increase the accumulation of lycopene in the T_0_ generation of tomato.

### Special Apparent Color Traits of Lycopene Mutant Fruits After the Breaker Stage of Ripening

**FIGURE 5:**
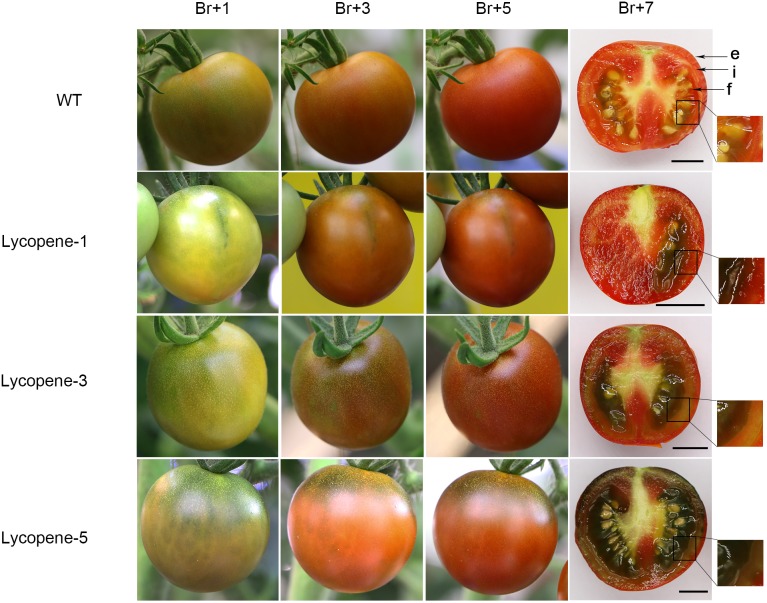
Phenotype of tomato fruit at different ripening stages. Three groups of transgenic tomato fruits were photographed at different times after the breaker stage of ripening and compared with that in WT. Sections were obtained at Br+7. Each group photograph was obtained from the same tomato fruit. e, exocarp; i, endocarp and f, flesh of the tomato fruit.

During the growth of lycopene mutants, most mutants in which the *SGR1* gene was targeted (alone or along with other genes) showed the desired rust color in fruit after the breaker stage of ripening (**Figure 5**). The *SGR1* gene silencing in tomato fruit has been shown to lead to the development of rust color containing both red and green (Luo et al., 2013). In Lycopene-1 and Lycopene-5, the differences in the cross-section of lycopene mutant fruits between Br+7 and Br+14 could be clearly observed (Supplementary Figure S5). The red portion of the entire surface of the fruit of Lycopene-1 and Lycopene-5 at Br+14 was significantly increased compared with that at Br+7, when the green portion was correspondingly reduced. Therefore, the green color in the endocarp of the fruit at Br+7 refers to the immature stage. However, the flesh color of Lycopene-5 was still green rather than red at Br+14. This residual green was distinct from that noted in the previous RNAi-SGR1 lines, but was similar to the apparent trait of the *SGR1* natural mutant (Luo et al., 2013). Thus, CRISPR/Cas9 specific-site DNA editing is more complete and comprehensive than the traditional RNAi technology of RNA silencing.

Interestingly, we also found a clear trend: higher was the number of genome sites that were modified in plants including *SGR1* gene targeted, the greater was the green portion in the cross-section of the fruit. Lycopene-1, Lycopene-3, and Lycopene-5 fruit cross-sections are shown in **Figure 5**. We found that the surface color of lycopene mutant fruits was significantly different from that of the WT fruits after the breaker stage of ripening; the green portion was clearly visible in the former. Further, the green colouration was more obviously closer to the stalk. In the same growth stage, the proportion of green part of fruit of Lycopene-1, Lycopene-3, and Lycopene-5 increased in turn. These results indicate that the combination of different target editing types affects the surface color of the tomato fruit.

### Segregation Patterns of CRISPR/Cas9-Medicated Targeted Mutagenesis

In order to obtain tomato varieties with high lycopene content and stable genetic traits, we selected the progeny of two lines to investigate the transmission pattern of CRISPR/Cas9-mediated mutations. As expected, Lycopene-1 T_1_ generation of targets T1-SGR1 and T2-SGR1 homozygotes were homozygous for the same mutations (**Table 1**), indicating that the mutations in the homozygotes were stably transferred to the next generation in a Mendelian fashion, which was consistent with the findings of previous studies (Feng et al., 2014; Zhang et al., 2014; Pan et al., 2016). However, an unexpected segregation ratio of 1:4:1 was observed in the T_1_ generation of the target T3 (*LCY-E*) lycopene-5 lines. These results indicated that two alleles in one biallelic mutant might not be inherited with equal frequencies (Xu et al., 2015). However, the segregation patterns of most heterozygotes and bialleles were less predictable, and many new mutants were found in the T_1_ lines, which might be attributed to somatic mutations. Studies in Arabidopsis have shown that most mutations in early generations are somatic mutations, leading to the difficulty in predetermining the targeted genotype in the subsequent generation (Jiang et al., 2013; Jacobs et al., 2015; Xu et al., 2015).

**Table 1:** Segregation patterns of CRISPRCas9-medicated targeted mutagenesis during the T_0_ to T_1_ generation.

Line^#^	Gene	Target	T_0_		T_1_		
				
			Zygosity^$^	Genotype	Number of read	Mutation segregation	Genotype
Lycopene-1	*SGR1*	T1	Heterozygote	wt,i1	21	7wt,9He,3B,2Ho	7wt;7He:wt,i1;1He:wt,d16;1He:wt,d2;3B:i1,i1;2Ho:i1i1
	*SGR1*	T2	Biallele	d1,i1		11B,10Ho	10B:d1i1;1B:i1i2;4Ho:i1i1;6Ho:d1d1
Lycopene-5	*SGR1*	T1	Homozygote	d1,d1	6	6Ho	6Ho:d1,d1
	*SGR1*	T2	Homozygote	d1,d1		6Ho	6Ho:d1,d1
	*LCY-E*	T3	Heterozygote	wt,d8		1wt,4He,1Ho	1wt;4He:wt,d8;1Ho:d8,d8
	*Blc*	T4	Biallele	i1,i1		4B, 2Ho	1B:d49,i1;1B:i1,i2;2B:i1,i1;2Ho:i1,i1
	LCY-B2	T6	Heterozygote	wt,i1		3wt,2He,1Ho	3wt;1He:wt,i1;1He:wt,d146;1Ho:i1i1


*^#^Line name is in the format of Lycopene-#. ^$^The zygosoty of homozygote, heterozygote and biallele in T_*0*_ plant lines were putative. d#, # of bp deleted at the target sites; i#, # number of bases insertion at target sites; wt, wild-type sequence without mutations detected at target sites.*

## Discussion

Multiplex CRISPR/Cas9 genome editing has been used for efficiently editing targets in various species (Miao et al., 2013; Brooks et al., 2014; Feng et al., 2014; Zhang et al., 2014; Fan et al., 2015; Ma et al., 2015; Xu et al., 2015). In this study, all the T_0_ lycopene mutants were genetically modified (Supplementary Figure S1), which confirms that the CRISPR/cas9 system has a high editing efficiency for well-designed sites at genes of interest in tomato. Ma et al. (2015) suggested that the GC contents and sgRNA secondary structure are important for improving target editing efficiency. Since, the GC content and base pair number (between sgRNA sequence and target sequence) were not significantly different among six target sites (Supplementary Table S1) in this study based on strict optimisation. Therefore, the distinct mutant efficiencies among tomato plants could likely be attributed to different promoters. AtU3b, AtU3d, AtU6-1, AtU6-29 are four different snRNA promoters cloned from Arabidopsis thaliana to avoid HR between sgRNA expression cassettes in Agrobacterium or plant genome (Ma et al., 2015). Specifically, there is a difference in length between them. AtU3b promoter is the longest, up to 344 bp; AtU3d promoter is the shortest, only 102 bp. The results in this study suggest that there may be a distinction in the efficiency of target editing between different promoter, but the exact mechanism is not clear. Unfortunately, none of the indel mutations were detected at the T5 target in all transgenic plants obtained. We speculated two reasons for this. One is the failure of gene targeting because of a series of complex and unclear mechanisms. The other is probably that the gene *Blc* selected by the T5 target has a very important role in the conversion of lycopene to α- and β-carotenoids. Mutation of this gene might produce lethal phenotypes, but the specific mechanism is not yet clear. In addition, target-sgRNAs with extended nucleotides at the 5′ end (derived from the vector ligation site) have been shown to guide genome editing in plants (Xie and Yang, 2013). Studies in rice suggest that this target-sgRNAs do not affect the editing efficiency (Ma et al., 2015). However, in this study, target T5 that did not show target editing and target T6 with significantly lower editing efficiency were so-called regular targets (using the U6 promoter, while the target 5′ end for G); whether this kind of target-sgRNAs affects the editing efficiency in tomato is not yet known.

To improve yield and stress resistance, researchers are paying considerable attention to the enhancement fruit quality of tomato. The content of lycopene, which is a kind of carotenoid, is one of the most important quality traits of tomato fruit, with industrial, health, and nutritional attributes. Since the amounts of fruit volatiles are correlated to carotenoid levels, the higher the lycopene content in a fruit, the more essential flavor volatiles are generated, rendering the fruit to be more nutritious (Klee and Giovannoni, 2011). The high demand for natural lycopene to serve as healthy antioxidant requires that tomato plants are converted to green factories for the economical production of high-value lycopene; this has triggered increasing interest in the up-regulation of lycopene content in fruit. In this study, we successfully enhanced lycopene in tomato fruits by using multiplex CRISPR/Cas9 genome editing. However, with an increase in mutant genes, no concomitant increase in lycopene content was noted in the lycopene mutants (**Figure 3C**); this could be speculated by two reasons: bottom-up of the carotenoid accumulation model associated with carotenoid metabolism in tomato (Luo et al., 2013) and the mechanism of the feedback regulation of the carotenoid metabolic pathway. However, it is obvious that whether such a feedback mechanism really works or not remains to be verified. The transformation pathway of lycopene to α-carotene and β-carotene was inhibited in groups Lycopene-3–5, unlike in groups in which only the *SGR1* gene was targeted (Lycopene-1). Therefore, the accumulation of the two above mentioned carotenoids and thus that of lycopene was affected in the tomato fruit. Further, we concluded that the promotion of lycopene synthesis may be better than the inhibition of its cyclisation to enhance the accumulation of lycopene by regulating the carotenoid metabolism networks in tomato.

The green coloration in the fruits of some lycopene mutants was mainly caused by target editing of the gene *SGR1* (**Figure 5**). *SGR* is a positively regulated target of *RIN*, and its proteins have been shown to play a critical role in the initiation of chlorophyll degradation and senescence (Jiang et al., 2007; Ren et al., 2007; Barry et al., 2008; Hortensteiner, 2009; Fujisawa et al., 2013). We found that the green color in the fruit surface of lycopene mutants would gradually weaken with time; however, some green residues remained within the fruit compared to that in WT. The reason for this phenomenon is not yet clear. Furthermore, previous studies suggested that the use of RNAi to silence the *SGR1* gene did not affect the maturation of tomato fruit. The time required for transgenic fruit diameter to reach 1 cm was reported to be not significantly different from that in WT (Luo et al., 2013). Thus, the combination of a gene or many genes such as the four genes (*SGR1*, *LCY-E*, *Blc*, and *LCY-B2*) might have directly or indirectly affected the maintenance of the tomato fruit transitions in color.

More recently, studies have shown that the CRISPR/cas9 system can be used for genome editing of transcription factors (Ito et al., 2015) and PDS as the reporter gene (Pan et al., 2016) in the model plant tomato. In addition, the CRISPR/cas9 system has also recently been used to enhance tomato fruit quality, resistance to disease, and to extend the shelf life of tomato plants. The content of γ-aminobutyric acid in tomato fruit has been reported to be effectively increased by CRISPR/cas9 (Li et al., 2017; Nonaka et al., 2017). Nekrasov et al. (2017) obtained tomato plants with stronger resistance to the powdery mildew fungal pathogen by using the CRISPR/Cas9 technology. Recent studies have also shown that CRISPR/Cas9-induced gene replacement via homology-directed repair provided a valuable method for generating tomato lines having a long shelf life (Yu et al., 2017). In this study, the pYLCRISPR/Cas9 multi-target editing system was successfully applied to create mutations of many related genes in carotenoid synthesis and metabolic pathways in tomato plants by artificially introducing a transformation vector containing multiple sgRNA expression cassettes to increase the content of lycopene in fruit. This study provides the basis for acquiring new tomato varieties with improved agricultural traits.

## Author Contributions

XL and HZ designed the research. YW and SC provided help for carotenoid analysis using HPLC. XL performed the experiments and drafted the manuscripts. HT, DF, BZ, and YL provided materials and intellectual input for the work.

## Conflict of Interest Statement

The authors declare that the research was conducted in the absence of any commercial or financial relationships that could be construed as a potential conflict of interest.

## Footnotes

1 http://cbi.hzau.edu.cn/crispr/

2 http://mfold.rna.albany.edu/?q=mfold/RNA-Folding-Form2.3

3 http://skl.scau.edu.cn/dsdecode/

4 http://skl.scau.edu.cn/targetdesign/

5 http://www.ebi.ac.uk/interpro/scan.html

## Abbreviations

AC: Ailsa Craig
Br+7: 7 days after breaker stage of ripening
CRISPR/Cas9: clustered regularly interspaced palindromic repeat associated protein 9
CRTISO: carotenoid isomerase
DMAPP: dimethylallyl diphosphate
DPA: days post anthesis
DSB: double-strand breaks
DXS: 1-deoxy-d-xylulose 5-phosphate synthase
G3P: glyceraldehyde 3-phosphatephytoene desaturase
GGPP: geranylgeranyl pyrophosphate
GGPPS: geranylgeranyl pyrophosphate synthase
HR: homologous recombination
indels: insertions or deletions
IPP: isopentenyl diphosphate
LCY-B: lycopene β-cyclase
LCY-E: lycopene δ-cyclase
MCR: mutagenic chain reaction
NHEJ: non-homologous end joining
PAM: protospacer-adjacent motif
PDS: phytoene desaturase
PSY1: phytoene synthase 1
RP-HPLC: reverse phase high-performance liquid chromatography
(s)gRNA: (single) guide RNA
TALEN: transcription activator-like effector nuclease
TEM: transmission electron microscopy
WT: wild type
ZFN: zinc finger nuclease
ZISO: z-carotene isomerase
