# Supplementary material for: Lycopene Is Enriched in Tomato Fruit by CRISPR/Cas9-Mediated Multiplex Genome Editing
Source: Front Plant Sci. 2018 Apr 26;9:559. doi: 10.3389/fpls.2018.00559 (PMC5935052; doi:10.3389/fpls.2018.00559)
Supplement: Supplementary file 1 [file Data_Sheet_1.DOC]

**Table S1 Details of target sequence in CRISPR/Cas9 binary expression cassette**

| Gene | Locus | sgRNA | Target sequence | PAM sequence | GC (%) | Promoter | pairing with gRNA(bp) |
| --- | --- | --- | --- | --- | --- | --- | --- |
| *SGR1* | Solyc08g080090 | T1 | GGCCTCCACTAATGTGGCAA | TGG | 55 | AtU3d | 5 |
|  |  | T2 | CCCCAGTGAGTGTTATGCCT | TGG | 55 | AtU3d | 4 |
| *LCY-E* | Solyc12g008980 | T3 | TTGGCACACGGAAGAATGCG | CGG | 55 | AtU3b | 5 |
| *Blc* | Solyc10g079480 | T4 | CTGGAAGGGGGCCTCCCATT | GGG | 65 | AtU3b | 5 |
| *LCY-B1* | Solyc04g040190 | T5 | GCCACCATATAACCGGTGGA | TGG | 55 | AtU6-1 | 5 |
| *LCY-B2* | Solyc06g074240 | T6 | GAGCTGGCCCTGCTGGGCTC | AGG | 75 | AtU6-29 | 5 |

Pairing with gRNA, the number of base pair (bp) that the targets pairing with gRNA.

| **Table S2 Primers used for recombinant pYLCRISPR/Cas9 vector construction** | | |
| --- | --- | --- |
| PCR | Primer | Sequence (5’-3’) |
| 1^st^ PCR | U-F | CTCCGTTTTACCTGTGGAATCG |
|  | gR-R | CGGAGGAAAATTCCATCCAC |
|  | AtU3d-T1SGR1 Rev | TTGCCACATTAGTGGAGGCCTGACCAATGGTGCTTTG |
|  | gR-T1SGR1 For | GGCCTCCACTAATGTGGCAAGTTTTAGAGCTAGAAAT |
|  | AtU3d-T2SGR1 Rev | AGGCATAACACTCACTGGGGTGACCAATGGTGCTTTG |
|  | gR-T2SGR1 For | CCCCAGTGAGTGTTATGCCTGTTTTAGAGCTAGAAAT |
|  | AtU3b-T3LCY-E Rev | CGCATTCTTCCGTGTGCCAATGACCAATGTTGCTCC |
|  | gR-T3LCY-E For | TTGGCACACGGAAGAATGCGGTTTTAGAGCTAGAAAT |
|  | AtU3b-T4Blc Rev | AATGGGAGGCCCCCTTCCAGTGACCAATGTTGCTCC |
|  | gR-T4Blc For | CTGGAAGGGGGCCTCCCATTGTTTTAGAGCTAGAAAT |
|  | AtU6-1-T5LCY-B1 Rev | TCCACCGGTTATATGGTGGCAATCACTACTTCGTCT |
|  | gR-T5LCY-B1 For | CCACCATATAACCGGTGGAGTTTTAGAGCTAGAAAT |
|  | AtU6-29-T6LCY-B2 Rev | GAGCCCAGCAGGGCCAGCTCAATCTCTTAGTCGACT |
|  | gR-T6LCY-B2 For | AGCTGGCCCTGCTGGGCTCGTTTTAGAGCTAGAAAT |
| 2^nd^ PCR | Pps-GGL | TTCAGAggtctcTctcgACTAGTATGGAATCGGCAGCAAAGG |
|  | Pgs-GG2 | AGCGTGggtctcGtcagggTCCATCCACTCCAAGCTC |
|  | Pps-GG2 | TTCAGAggtctcTctgacacTGGAATCGGCAGCAAAGG |
|  | Pgs-GG3 | AGCGTGggtctcGtcttcacTCCATCCACTCCAAGCTC |
|  | Pps-GG3 | TTCAGAggtctcTaagacttTGGAATCGGCAGCAAAGG |
|  | Pgs-GG4 | AGCGTGggtctcGagtccttTCCATCCACTCCAAGCTC |
|  | Pps-GG4 | TTCAGAggtctcTgactacaTGGAATCGGCAGCAAAGG |
|  | Pgs-GG5 | AGCGTGggtctcGgtccacaTCCATCCACTCCAAGCTC |
|  | Pps-GG5 | TTCAGAggtctcTggacttgTGGAATCGGCAGCAAAGG |
|  | Pgs-GG6 | AGCGTGggtctcGcagatagTCCATCCACTCCAAGCTC |
|  | Pps-GG6 | TTCAGAggtctcTtctgcaaTGGAATCGGCAGCAAAGG |
|  | Pgs-GGR | AGCGTGggtctcGaccgACGCGTATCCATCCACTCCAAGCT |

| **Table S3 Primers used for target site mutation analysis** | | |  |
| --- | --- | --- | --- |
| Target | Primer | Sequence (5’-3’) | Size of PCR products |
| T1SGR1,  T2SGR1 | T1-For | GCTCATGACGCATGTCGAAATC | 1080bp |
|  | T2-Rev | GGCACAACCCAACTTACAATAATTG |  |
|  | T1-Seq | GTAGAACTTCTGTTGAGAAAAGTGG |  |
|  | T2-Seq | CAGAGATGCAACTTCCCCCTC |  |
| T3LCY-E | T3-For | CTTCAAAAATGCCACTGGACGC | 448bp |
|  | T3-Rev | CATGTTGTAAGATGTTGCCTGCCTC |  |
|  | T3-Seq | CAGCTTGGAACACTCTTTGGCC |  |
| T4Blc | T4-For | GGCATATTGGCACAAGTGGAGG | 450bp |
|  | T4-Rev | CCTTCTTGTAGCGGATAAATCAAGC |  |
|  | T4-Seq | GCCATGCCATTTTCATCAAACAG |  |
| T5LCY-B1 | T5-For | CAATGCCATTTTCATCCAACAGG | 493bp |
|  | T5-Rev | CCATGCCAATAACGAGGTTCTAAG |  |
|  | T5-Seq | CGAATGGTGGCTCGTTTAAACC |  |
| T6LCY-B2 | T6-For | GGAAACTCTTCTCAAGCCTTTTCC | 440bp |
|  | T6-Rev | GCACACAAGTCATAGGCCATTTATG |  |
|  | T6-Seq | GCACCCACATCAAAGCCAGAG |  |

T_#_-For and T_#_-Rev were the forward and reverse primers for PCR amplification of each target, respectively. T_#_-Seq was the primers used to sequence the corresponding PCR products.

| **Table S4** **Primers used for off-target site mutation analysis** | | |  |
| --- | --- | --- | --- |
| Target | Primer | Sequence (5’-3’) | Size of PCR products |
| T1SGR1 | OFF1-For | GTCGTAACGCAAGCATTAGTGG | 185bp |
|  | OFF1-Rev | GCGAGTCCACTTATCTCATTAACC |  |
|  | OFF2-For | CAGCTTACCGCAGGAAGTGTTC | 459bp |
|  | OFF2-Rev | CTGATTAGAGCTTCTCCCGAATACC |  |
| T2SGR1 | OFF3-For | GCAGTGCCAAGGACATAGTCAGG | 428bp |
|  | OFF3-Rev | CGAAAACCCCTAAGTCACTAGGACC |  |
|  | OFF4-For | CTTGGAGAAAGATCAGCCAATTCC | 462bp |
|  | OFF4-Rev | GTAGGTTCCTTTACCCAAAAGGGTG |  |
| T3LCY-E | OFF5-For | GGCCATACCAATATGTTTGTTGTG | 460bp |
|  | OFF5-Rev | GAGTTCAATGGCGAACTCGATG |  |
|  | OFF6-For | GGGAAAACAACAAGAGAAGCTGG | 428bp |
|  | OFF6-Rev | CAACCCAATACATTCAGCTCGAG |  |
| T4Blc | OFF7-For | GAGAGAGGTGCTAGTGTGCCTTAGC | 494bp |
|  | OFF7-Rev | CATCATAAAATAGCCCTTCGAGCAG |  |
|  | OFF8-For | CGTCGAGGAACAAGTCCATGTC | 412bp |
|  | OFF8-Rev | GTCTGTGGATACTTCCTAAACGAGG |  |
| T6LCY-B2 | OFF9-For | GGAAAGATAGGGAAAGAACGGGG | 435bp |
|  | OFF9-Rev | CCCACACACCAATTTGATTCAATTC |  |
|  | OFF10-For | GTGGTTTTGGTTCGTGTCTCTG | 426bp |
|  | OFF10-Rev | GGACTCAAGGATCATGATCTTGTG |  |

OFF_#_-For and OFF_#_-Rev were the forward and reverse primers for PCR amplification of each target, respectively. OFF_#_-For was used to sequence the corresponding PCR products.

**Table S5 Mutation rates in T_0_ plants with each 2 sites among all targets**

| Site1 | Site2 | No. of T0 plants examined | No. of plants with mutations | | | No. of plants with mutations (%) | | | Expected double mutation frequency (%)^※^ |
| --- | --- | --- | --- | --- | --- | --- | --- | --- | --- |
|  |  |  | site1 | site2 | Both sites | site1 | site2 | Both sites |  |
| T1 | T2 | 24 | 10 | 23 | 10 | 41.67 | 95.83 | 41.67 | 39.93 |
| T1 | T3 | 24 | 10 | 2 | 1 | 41.67 | 8.33 | 4.17 | 3.47 |
| T1 | T4 | 24 | 10 | 22 | 9 | 41.67 | 91.67 | 37.50 | 38.19 |
| T1 | T5 | 24 | 10 | 0 | 0 | 41.67 | 0.00 | 0.00 | 0.00 |
| T1 | T6 | 24 | 10 | 1 | 1 | 41.67 | 4.17 | 4.17 | 1.74 |
| T2 | T3 | 24 | 23 | 2 | 2 | 95.83 | 8.33 | 8.33 | 7.99 |
| T2 | T4 | 24 | 23 | 22 | 21 | 95.83 | 91.67 | 87.50 | 87.85 |
| T2 | T5 | 24 | 23 | 0 | 0 | 95.83 | 0.00 | 0.00 | 0.00 |
| T2 | T6 | 24 | 23 | 1 | 4 | 95.83 | 4.17 | 16.67 | 3.99 |
| T3 | T4 | 24 | 2 | 22 | 2 | 8.33 | 91.67 | 8.33 | 7.64 |
| T3 | T5 | 24 | 2 | 0 | 0 | 8.33 | 0.00 | 0.00 | 0.00 |
| T3 | T6 | 24 | 2 | 1 | 1 | 8.33 | 4.17 | 4.17 | 0.35 |
| T4 | T5 | 24 | 22 | 0 | 0 | 91.67 | 0.00 | 0.00 | 0.00 |
| T4 | T6 | 24 | 22 | 1 | 1 | 91.67 | 4.17 | 4.17 | 3.82 |
| T5 | T6 | 24 | 0 | 1 | 0 | 0.00 | 4.17 | 0.00 | 0.00 |

^※^ Expectes double mutation rate if mutation at each site is independent of each other (product of mutation rates at 2 individual sites).

| **Table S6 Primers used for analysis of Cas9 expression** | | |  |
| --- | --- | --- | --- |
| Gene | Primer | Sequence (5’-3’) | Size of PCR products |
| *Cas9* | Cas9- For | GAAGGTTGGTATTCACGGGG | 536bp |
|  | Cas9- Rev | TAAGGAAGTGACCACGGAAC |  |

Cas9-For and Cas9-Rev were the forward and reverse primers for PCR amplification of gene *Cas9*.

**Table S7** Mutation analyzed of potential off-target sites

| Target | Name of putative off-target sites | Putative off-target locus | Putative off-target sequence | No.of mismatch bases | No. of plants examined | No. of indel mutation |
| --- | --- | --- | --- | --- | --- | --- |
| T1 | OFF1 | ch12:62427413-62427435 | TTATTGTCACATTAGTGGAGACC | 3 | 5 | 0 |
|  | OFF2 | ch09:61714065-61714087 | GGACACTACAAATGTGGCAATAG | 4 | 5 | 0 |
| T2 | OFF3 | ch09:42845910-42845932 | GTTAGGCATGACACTCACTGGGG | 4 | 5 | 0 |
|  | OFF4 | ch07: 17871428-17871450 | CGTTAGTGAGTGCTATGCCTAGG | 4 | 5 | 0 |
| T3 | OFF5 | ch08:5794751-5794773 | TTAGCACTCTGAAGAATGGGTGG | 4 | 3 | 0 |
|  | OFF6 | ch10:18255441-18255463 | TTGGCAGAAGAAAGAATGGGAGG | 4 | 3 | 0 |
| T4 | OFF7 | ch03:43805825-43805847 | CTGTTAGGCGGCGTCCCATTGAG | 4 | 5 | 0 |
|  | OFF8 | ch05:44503964-44503986 | CTGTAGGAGGGCCTACCATTGGG | 4 | 5 | 0 |
| T6 | OFF9 | ch01:5935474-5935496 | GAGCATGCCCTGATGGGCTTTGG | 4 | 3 | 0 |
|  | OFF10 | ch08:37698871-37698893 | GAGGCTGCCCTGCGGGGCTCTGG | 4 | 3 | 0 |

PAM sequence (NGG) is indicated in blue. Mismatch nucleotides are marked in red.
