# Supplementary material for: Lycopene Is Enriched in Tomato Fruit by CRISPR/Cas9-Mediated Multiplex Genome Editing
Source: Front Plant Sci. 2018 Apr 26;9:559. doi: 10.3389/fpls.2018.00559 (PMC5935052; doi:10.3389/fpls.2018.00559)
Supplement: Supplementary file 2 [file Image_1.PDF]

Lycopene-1

Editing type of CR-lycopene-3

T2	WT	TGTTGCTTTCCCCCAGTGAGTGTTATGCCTTGGCTTTCTT	
CR-lycopene-3	Allele1	TGTTGCTTTCCCCCAGTGAGTGTTATGCCTTGGCTTTCTT	(wild type)
(Heterzygous)	Allele2	TGTTGCTTTCCCCCAGTGAGTGTTAT-CCTTGGCTTTCTT	(1bp deletion)

Editing type of CR-lycopene-10

T1	WT	TTCATGTCCATTG-CCACATTAGTGGAGGCCATTTTATGTT	
CR-lycopene-10	Allele1	TTCATGTCCATTG-CCACATTAGTGGAGGCCATTTTATGTT	(wild type)
(Heterzygous)	Allele2	TTCATGTCCATTGTCCACATTAGTGGAGGCCATTTTATGTT	(1bp insertion)
T2	WT	TGTTGCTTTCCCCCAGTGAGTGTTATG-CCTTGGCTTTCTT	
CR-lycopene-10	Allele1	TGTTGCTTTCCCCCAGTGAGTGTTAT--CCTTGGCTTTCTT	(1bp deletion)
(Biallelic)	Allele2	TGTTGCTTTCCCCCAGTGAGTGTTATGCCCTTGGCTTTCTT	(1bp insertion)

Lycopene-2

Editing type of CR-lycopene-18

T4	WT	GTGTGATCCCAATGGGAGGCCCCCTTCCAGTAATACCTCA	
CR-lycopene-18	Allele1	GTGTGATCCCAAT-------CCCCTTCCAGTAATACCTCA	(7bp deletion)
(Biallelic)	Allele2	GTGTGATCCCAAT----GGCCCCCTTCCAGTAATACCTCA	(4bp deletion)

Lycopene-3

Editing type of CR-lycopene-1

T2	WT	TGTTGCTTTCCCCCAGTGAGTGTTATG-CCTTGGCTTTCTT	
CR-lycopene-1	Allele1	TGTTGCTTTCCCCCAGTGAGTGTT----CCTTGGCTTTCTT	(3bp deletion)
(Biallelic)	Allele2	TGTTGCTTTCCCCCAGTGAGTGTTATGACCTTGGCTTTCTT	(1bp insertion)
T4	WT	GTGTGATCCCAATGGGAGGCCCCCTTCCAGTAATACCTCA	
CR-lycopene-1	Allele1	GTGTGATCCCAATGGGAGGCCCCCTTCCAGTAATACCTCA	(wild type)
(Heterzygous)	Allele2	GTGTGATCCCAAT----GGCCCCCTTCCAGTAATACCTCA	(4bp deletion)

Editing type of CR-lycopene-2

T2	WT	TGTTGCTTTCCCCCAGTGAGTGTTATGCCTTGGCTTTCTT	
CR-lycopene-2	Allele1	TGTTGCTTTCCCCCAGTGAGTGTTAT-CCTTGGCTTTCTT	(1bp deletion)
(Homozygous)	Allele2	TGTTGCTTTCCCCCAGTGAGTGTTAT-CCTTGGCTTTCTT	(1bp deletion)
T4	WT	GTGTGATCCCAATGGGAGGCCCCCTTCCAGTAATACCTCA	
CR-lycopene-2	Allele1	GTGTGATCCCAATGGGAGGCCCCCTTCCAGTAATACCTCA	(wild type)
(Heterzygous)	Allele2	GTGTGATCCCAAT--GAGGCCCCCTTCCAGTAATACCTCA	(2bp deletion)

Editing type of CR-lycopene-4

T2	WT	TGTTGCTTTCCCCCAGTGAGTGTTATGCCTTGGCTTTCTT	
CR-lycopene-4	Allele1	TGTTGCTTTCCCCCAGTGAGTGTTAT-CCTTGGCTTTCTT	(1bp deletion)
(Homozygous)	Allele2	TGTTGCTTTCCCCCAGTGAGTGTTAT-CCTTGGCTTTCTT	(1bp deletion)
T4	WT	GTGTGATCCCAATGGGAGGCCCCCTTCCAGTAATACCTCA	
CR-lycopene-4	Allele1	GTGTGATCCCAAT----GGCCCCCTTCCAGTAATACCTCA	(4bp deletion)
(Biallelic)	Allele2	------------ACCAAGTCACTT----------------	(77bp deletion and 12bp substitution)

Editing type of CR-lycopene-5

T2	WT	TGTTGCTTTCCCCCAGTGAGTGTTATGCCTTGGCTTTCTT	
CR-lycopene-5	Allele1	TGTTGCTTTCCCCCAGTGAGTGTTAT-CCTTGGCTTTCTT	(1bp deletion)
(Homozygous)	Allele2	TGTTGCTTTCCCCCAGTGAGTGTTAT-CCTTGGCTTTCTT	(1bp deletion)
T4	WT	GTGTGATCCCAATGGGAGGCCCCCTTCCAGTAATACCTCA	
CR-lycopene-5	Allele1	GTGTGATCCCAAT----GGCCCCCTTCCAGTAATACCTCA	(4bp deletion)
(Biallelic)	Allele2	------------AAGTGACTTGGT----------------	(80bp deletion and 12bp substitution)

Editing type of CR-lycopene-6

T1	WT	TTCATGTCCATTGCCACATTAGTGGAGGCCATTTTATGTT	
CR-lycopene-6	Allele1	TTCATGTCCA---CCACATTAGTGGAGGCCATTTTATGTT	(3bp deletion)
(Biallelic)	Allele2	TTCATGTCCAT-GCCACATTAGTGGAGGCCATTTTATGTT	(1bp deletion)
T2	WT	TGTTGCTTTCCCCCAGTGAGTGTTATG-CCTTGGCTTTCTT	
CR-lycopene-6	Allele1	TGTTGCTTTCCCCCAGTGAGTGTTATG-CCTTGGCTTTCTT	(wild type)
(Heterzygous)	Allele2	TGTTGCTTTCCCCCAGTGAGTGTTATGACCTTGGCTTTCTT	(1bp insertion)
T4	WT	GTGTGATCCCAATGGGAGGCCCCCTTCCAGTAATACCTCA	
CR-lycopene-6	Allele1	GTGTGATCCCAATGGGAGGCCCCCTTCCAGTAATACCTCA	(wild type)
(Heterzygous)	Allele2	------------CGCAGCTCCTGTGAAGC-----------	(289bp deletion and 17bp substitution)

Editing type of CR-lycopene-7

T2	WT	TGTTGCTTTCCCCCAGTGAGTGTTATGCCTTGGCTTTCTT	
CR-lycopene-7	Allele1	TGTTGCTTTCCCCCAGTGAGTGTTAT-CCTTGGCTTTCTT	(1bp deletion)
(Homozygous)	Allele2	TGTTGCTTTCCCCCAGTGAGTGTTAT-CCTTGGCTTTCTT	(1bp deletion)
T4	WT	GTGTGATCCCAATGGGAGGCCCCCTTCCAGTAATACCTCA	
CR-lycopene-7	Allele1	GTGTGATCCCAAT----GGCCCCCTTCCAGTAATACCTCA	(4bp deletion)
(Biallelic)	Allele2	------------AAGTGACTTGGT----------------	(80bp deletion and 12bp substitution)

Editing type of CR-lycopene-8

T2	WT	TGTTGCTTTCCCCCAGTGAGTGTTATGCCTTGGCTTTCTT	
CR-lycopene-8	Allele1	TGTTGCTTTCCCCCAGTGAGTGTTAT-CCTTGGCTTTCTT	(1bp deletion)
(Homozygous)	Allele2	TGTTGCTTTCCCCCAGTGAGTGTTAT-CCTTGGCTTTCTT	(1bp deletion)
T4	WT	GTGTGATCCCAATGGGAGGCCCCCTTCCAGTAATACCTCA	
CR-lycopene-8	Allele1	GTGTGATCCCAAT----GGCCCCCTTCCAGTAATACCTCA	(4bp deletion)
(Biallelic)	Allele2	------------ACCAAGTCACTT----------------	(77bp deletion and 12bp substitution)

Editing type of CR-lycopene-9

	T1 WT	TTCATGTCCATTGCCACATTAGTGGAGGCCATTTTATGTT	
CR-lycopene-9 (Homozygous)	T2 WT	TGTTGCTTTCCCCCAGTGAGTGTTATGCCTTGGCTTTCTT	

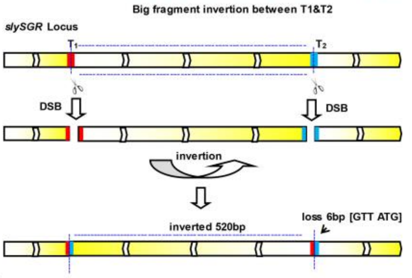

(520bp invertion)

T4	WT	GTGTGATCCCAATGGGAGGCCCCCTTCCAGTAATACCTCA	
CR-lycopene-9	Allele1	GTGTGATCCCAAT----GGCCCCCTTCCAGTAATACCTCA	(4bp deletion)
(Biallelic)	Allele2	--	(395bp deletion)

Editing type of CR-lycopene-11

T2	WT	TGTTGCTTTCCCCCAGTGAGTGTTATGCCTTGGCTTTCTT	
CR-lycopene-11	Allele1	TGTTGCTTTCCCCCAGTGAGTGTTAT-CCTTGGCTTTCTT	(1bp deletion)
(Homozygous)	Allele2	TGTTGCTTTCCCCCAGTGAGTGTTAT-CCTTGGCTTTCTT	(1bp deletion)
T4	WT	GTGTGATCCCAATGGGAGGCCCCCTTCCAGTAATACCTCA	
CR-lycopene-11	Allele1	GTGTGATCCCAAT----GGCCCCCTTCCAGTAATACCTCA	(4bp deletion)
(Biallelic)	Allele2	------------ACCAAGTCACTT -----------------	(77bp deletion and 12bp substitution)

Editing type of CR-lycopene-14

	T1	WT	TTCATGTCCATTGCCACATTAGTGGAGGCCATTTTATGTT
CR-lycopene-14	T2	WT	TGTTGCTTTCCCCCAGTGAGTGTTATGCCTTGGCTTTCTT
(Homozygous)			

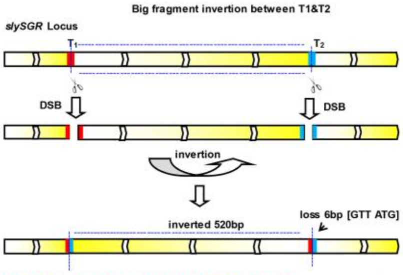

(520bp invertion)

T4	WT	GTGTGATCCCAATGGGAGGCCCCCTTCCAGTAATACCTCA	
CR-lycopene-14	Allele1	GTGTGATCCCAAT----GGCCCCCTTCCAGTAATACCTCA	(4bp deletion)
(Biallelic)	Allele2	--	(395bp deletion)

Editing type of CR-lycopene-15

T2	WT	TGTTGCTTTCCCCCAGTGAGTGTTATG-CCTTGGCTTTCTT	
CR-lycopene-15	Allele1	TGTTGCTTTCCCCCAGTGAGTGTTAT--CCTTGGCTTTCTT	(1bp deletion)
(Biallelic)	Allele2	TGTTGCTTTCCCCCAGTGAGTGTTATGTCCTTGGCTTTCTT	(1bp insertion)
T4	WT	GTGTGATCCCAATGGGAGGCCCCCTTCCAGTAATACCTCA	
CR-lycopene-15	Allele1	GTGTGATCCCAAT-GGAGGCCCCCTTCCAGTAATACCTCA	(1bp deletion)
(Biallelic)	Allele2	GTGTGATCCCAAT-TGAGGCCCCCTTCCAGTAATACCTCA	(1bp deletion and 1bp substitution)

Editing type of CR-lycopene-16

T1	WT	TTCATGTCCATTGCCACATTAGTGGAGGCCATTTTATGTT	
CR-lycopene-16	Allele1	TTCATGTCCATTGCCACATTAGTGGAGGCCATTTTATGTT	(wild type)
(Heterzygous)	Allele2	TTCATGTCCATTG-CACATTAGTGGAGGCCATTTTATGTT	(1bp deletion)
T2	WT	TGTTGCTTTCCCCCAGTGAGTGTTATG-CCTTGGCTTTCTT	
CR-lycopene-16	Allele1	TGTTGCTTTCCCCCAGTGAGTGTTAT--CCTTGGCTTTCTT	(1bp deletion)
(Biallelic)	Allele2	TGTTGCTTTCCCCCAGTGAGTGTTATGTCCTTGGCTTTCTT	(1bp insertion)
T4	WT	GTGTGATCCCAAT-GGGAGGCCCCCTTCCAGTAATACCTCA	
CR-lycopene-16	Allele1	GTGTGATCCCAAT--GGAGGCCCCCTTCCAGTAATACCTCA	(1bp deletion)
(Biallelic)	Allele2	GTGTGATCCCAATTGGGAGGCCCCCTTCCAGTAATACCTCA	(1bp insertion)

Editing type of CR-lycopene-17

T2	WT	TGTTGCTTTCCCCCAGTGAGTGTTATGCCTTGGCTTTCTT	
CR-lycopene-17	Allele1	TGTTGCTTTCCCCCAGTGAGTGTTATGCCTTGGCTTTCTT	(wild type)
(Chimeric)	Allele2	TGTTGCTTTCCCCCAGTGAGTG-----CCTTGGCTTTCTT	(5bp deletion)
	Allele3	TGTTGCTTTCCCCCAGTGAGTGT----CCTTGGCTTTCTT	(4bp deletion)
	Allele4	TGTTGCTTTCCCCCAGTGAGTGTTAT-CCTTGGCTTTCTT	(1bp deletion)
T4	WT	GTGTGATCCCAAT-GGGAGGCCCCCTTCCAGTAATACCTCA	
CR-lycopene-17	Allele1	GTGTGATCCCAAT--GGAGGCCCCCTTCCAGTAATACCTCA	(1bp deletion)
(Biallelic)	Allele2	GTGTGATCCCAATTGGGAGGCCCCCTTCCAGTAATACCTCA	(1bp insertion)

Editing type of CR-lycopene-19

Line	Target		Sequence	Editing
CR-lycopene-19 (Homozygous)	T1	WT	TTCATGTCCATTGCCACATTAGTGGAGGCCATTTTATGTT	
	T2	WT	TGTTGCTTTCCCCCAGTGAGTGTTATGCCTTGGCTTTCTT	

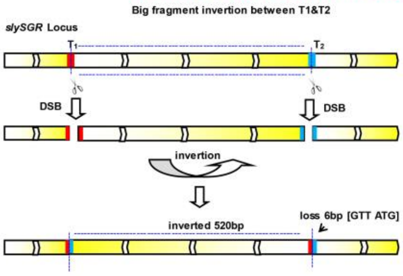

(520bp invertion)

Line	Target		Sequence	Editing
	T4	WT	GTGTGATCCCAATGGGAGGCCCCCTTCCAGTAATACCTCA	
CR-lycopene-19 (Biallelic)		Allele1	GTGTGATCCCAAT----GGCCCCCTTCCAGTAATACCTCA	(4bp deletion)
		Allele2	---------------------------------------	(395bp deletion)

Editing type of CR-lycopene-20

Line	Target		Sequence	Editing
	T1	WT	TTCATGTCCATTG-CCACATTAGTGGAGGCCATTTTATGTT	
CR-lycopene-20 (Biallelic)		Allele1	TTCATGTCCATTG---ACATTAGTGGAGGCCATTTTATGTT	(2bp deletion)
		Allele2	TTCATGTCCATTGTCCACATTAGTGGAGGCCATTTTATGTT	(1bp insertion)
	T2	WT	TGTTGCTTTCCCCCAGTGAGTGTTATG-CCTTGGCTTTCTT	
CR-lycopene-20 (Biallelic)		Allele1	TGTTGCTTTCCCCCAGTGAGTGTT----CCTTGGCTTTCTT	(3bp deletion)
		Allele2	TGTTGCTTTCCCCCAGTGAGTGTTATGTCCTTGGCTTTCTT	(1bp insertion)
	T4	WT	GTGTGATCCCAATGGGAGGCCCCCTTCCAGTAATACCTCA	
CR-lycopene-20 (Biallelic)		Allele1	GTGTGATCCCAAT-----------CTTCCAGTAATACCTCA	(1bp deletion)
		Allele2	----------------------------CAGTAATACCTCA	(42bp deletion)

Editing type of CR-lycopene-21

Line	Target		Sequence	Editing
	T1	WT	TTCATGTCCATTGCCACATTAGTGGAGGCCATTTTATGTT	
CR-lycopene-21 (Homozygous)		Allele1	TTCATGTCCATTG-CACATTAGTGGAGGCCATTTTATGTT	(1bp deletion)
		Allele2	TTCATGTCCATTG-CACATTAGTGGAGGCCATTTTATGTT	(1bp deletion)
	T2	WT	TGTTGCTTTCCCCCAGTGAGTGTTATGCCTTGGCTTTCTT	
CR-lycopene-21 (Homozygous)		Allele1	TGTTGCTTTCCCCCAGTGAGTGTTAT-CCTTGGCTTTCTT	(1bp deletion)
		Allele2	TGTTGCTTTCCCCCAGTGAGTGTTAT-CCTTGGCTTTCTT	(1bp deletion)
	T4	WT	GTGTGATCCCAAT-GGGAGGCCCCCTTCCAGTAATACCTCA	
CR-lycopene-21 (Biallelic)		Allele1	GTGTGATCCCAATTGGGAGGCCCCCTTCCAGTAATACCTCA	(1bp insertion)
		Allele2	GTGTGATCCCAATGGGGAGGCCCCCTTCCAGTAATACCTCA	(1bp insertion)

Editing type of CR-lycopene-22

Line	Target		Sequence	Editing
	T2	WT	TGTTGCTTTCCCCCAGTGAGTGTTATG-CCTTGGCTTTCTT	
CR-lycopene-22 (Homozygous)		Allele1	TGTTGCTTTCCCCCAGTGAGTGTTAT--CCTTGGCTTTCTT	(1bp deletion)
		Allele2	TGTTGCTTTCCCCCAGTGAGTGTTATGTCCTTGGCTTTCTT	(1bp insertion)
	T4	WT	GTGTGATCCCAATGGGAGGCCCCCTTCCAGTAATACCTCA	
CR-lycopene-22 (Biallelic)		Allele1	GTGTGATC-----GGGAGGCCCCCTTCCAGTAATACCTCA	(5bp deletion)
		Allele2	GTGTGATCCCAAT-GGAGGCCCCCTTCCAGTAATACCTCA	(1bp deletion)

Editing type of CR-lycopene-23

CR-lycopene-23 (Homozygous)

T1 WT TTCATGTCCATTGCCACATTAGTGGAGGCCATTTTATGTT
T2 WT TGTTGCTTTCCCCCAGTGAGTGTTATGCCTTGGCTTTCTT

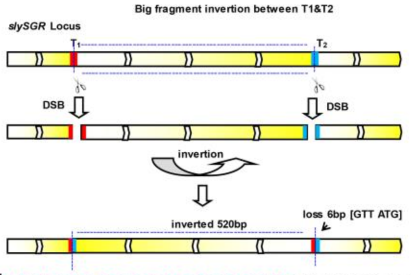

(520bp invertion)

T4 CR-lycopene-23 (Biallelic)

WT GTGTGATCCCAATGGGAGGCCCCCTTCCAGTAATACCTCA
Allele1 GTGTGATCCCAAT----GGCCCCCTTCCAGTAATACCTCA (4bp deletion)
Allele2 ------------------------------------- (395bp deletion)

Editing type of CR-lycopene-24

T2 CR-lycopene-24 (Biallelic)

WT TGTTGCTTTCCCCCAGTGAGTGTTATG-CCTTGGCTTTCTT
Allele1 TGTTGCTTTCCCCCAGTGAGTGTTAT--CCTTGGCTTTCTT (1bp deletion)
Allele2 TGTTGCTTTCCCCCAGTGAGTGTTATGTCCTTGGCTTTCTT (1bp insertion)

T4 CR-lycopene-24 (Biallelic)

WT GTGTGATCCCAAT-GGGAGGCCCCCTTCCAGTAATACCTCA
Allele1 GTGTGATCCCAAT--GGAGGCCCCCTTCCAGTAATACCTCA (1bp deletion)
Allele2 GTGTGATCCCAATTGGGAGGCCCCCTTCCAGTAATACCTCA (1bp insertion)

Lycopene-4

Editing type of CR-lycopene-13

T2 CR-lycopene-13 (Chimeric)

WT TGTTGCTTTCCCCCAGTGAGTGTTATG-CCTTGGCTTTCTT
Allele1 TGTTGCTTTCCCCCAGTGAGTGTTAT---CTTGGCTTTCTT (2bp deletion)
Allele2 TGTTGCTTTCCCCCAGTGAGTGTTAT--CCTTGGCTTTCTT (1bp deletion)
Allele3 TGTTGCTTTCCCCCAGTGAGTGTTATGACCTTGGCTTTCTT (1bp insertion)
Allele4 TGTTGCTTTCCTCCAGTGAGT-----------TTGGCTTTCTT (8bp deletion and 1bp substitution)

T3 CR-lycopene-13 (Heterzygous)

WT CATTTTTCCGCGCATTCTTCCGTGTGCCAAAATGGTATGG
Allele1 CATTTTTCCGCGCATTCTTCCGTGTGCCAAAATGGTATGG (wild type)
Allele2 CATTTTTCCGCGCA------CGTGTGCCAAAATGGTATGG (6bp deletion)

T4 CR-lycopene-13 (Chimeric)

WT GTGTGATCCCAATGGGAGGCCCCCTTCCAGTAATACCTCA
Allele1 GTGTGATCCCAATGGGAGGCCCCCTTCCAGTAATACCTCA (wild type)
Allele2 GTGTGATCCCAAT-----GCCCCCTTCCAGTAATACCTCA (5bp deletion)
Allele3 GTGTGATCCCAAT----GGCCCCCTTCCAGTAATACCTCA (4bp deletion)

Lycopene-5

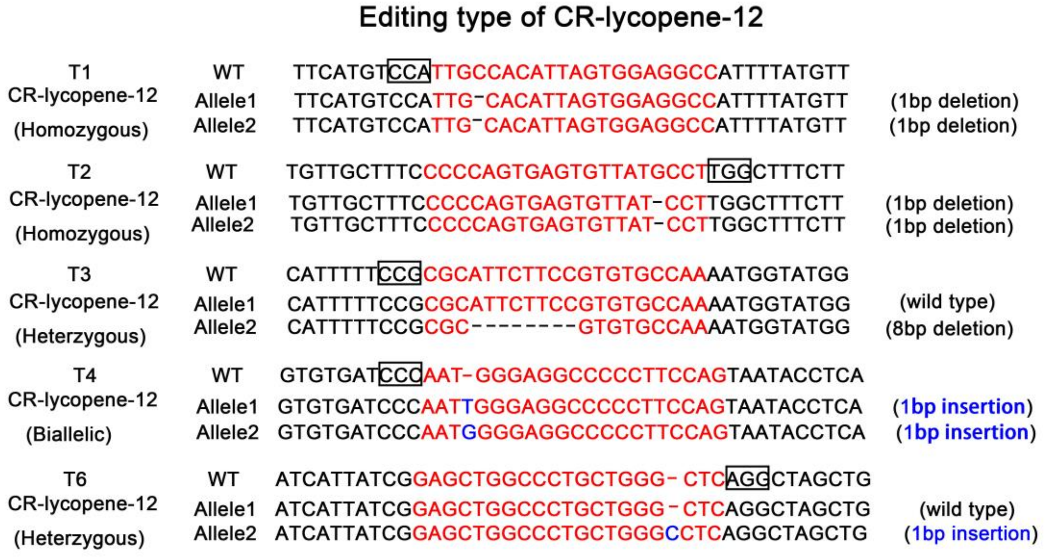

Figure S1 Specific mutations in each target of transgenic T_0 plants

The red type marks the target sequence, and the box represents the PAM motif. The blue type represents the base insert, the orange type shows the base substitution, and the black horizontal line indicates the base deletion. The specific results of inversion have been marked in the figure.
