# Supplementary figures and images for: Lycopene Is Enriched in Tomato Fruit by CRISPR/Cas9-Mediated Multiplex Genome Editing
Source: Front Plant Sci. 2018 Apr 26;9:559. doi: 10.3389/fpls.2018.00559 (PMC5935052; doi:10.3389/fpls.2018.00559)

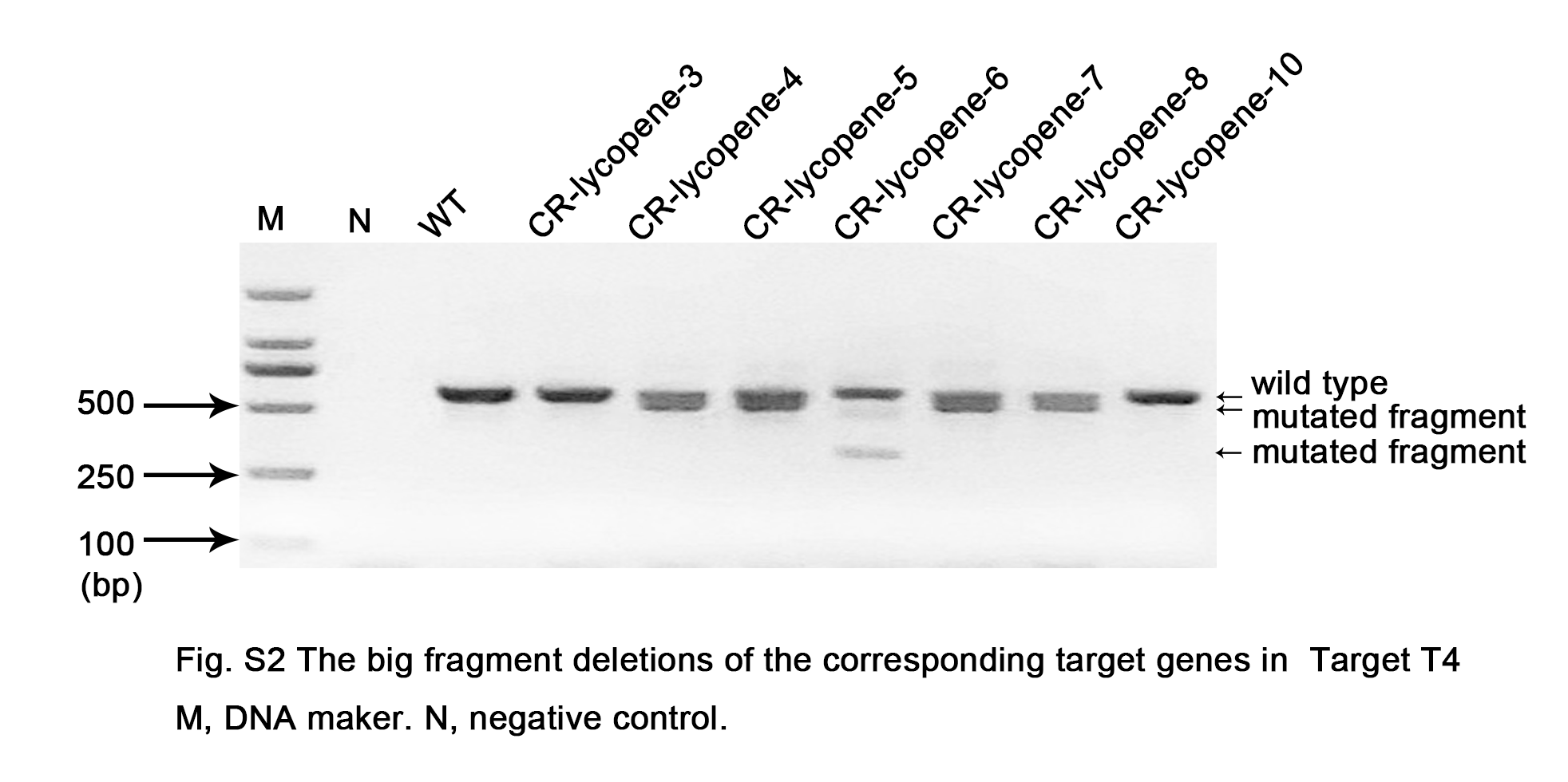

Supplement: Supplementary file 3 [file Image_2.TIF]

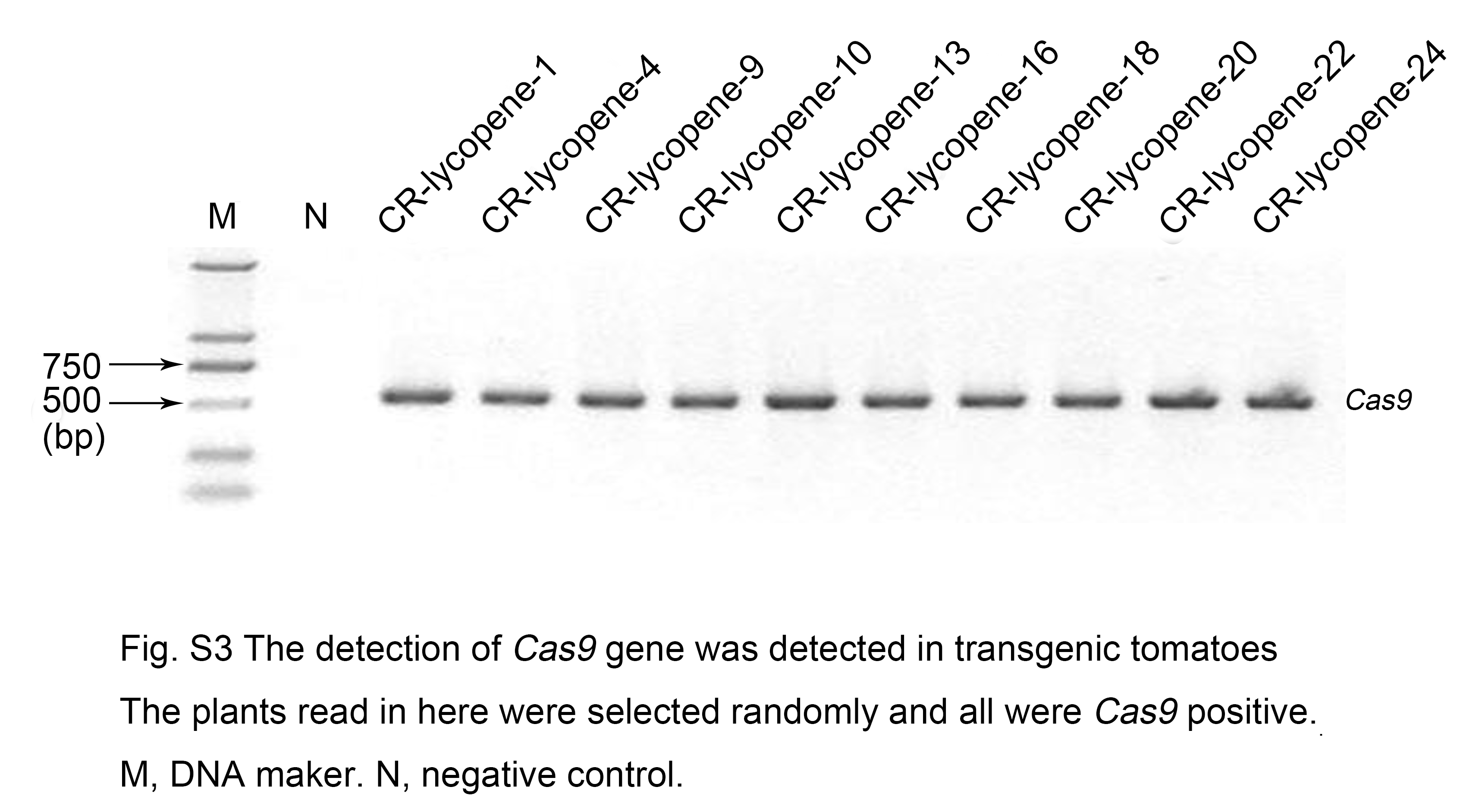

Supplement: Supplementary file 4 [file Image_3.TIF]

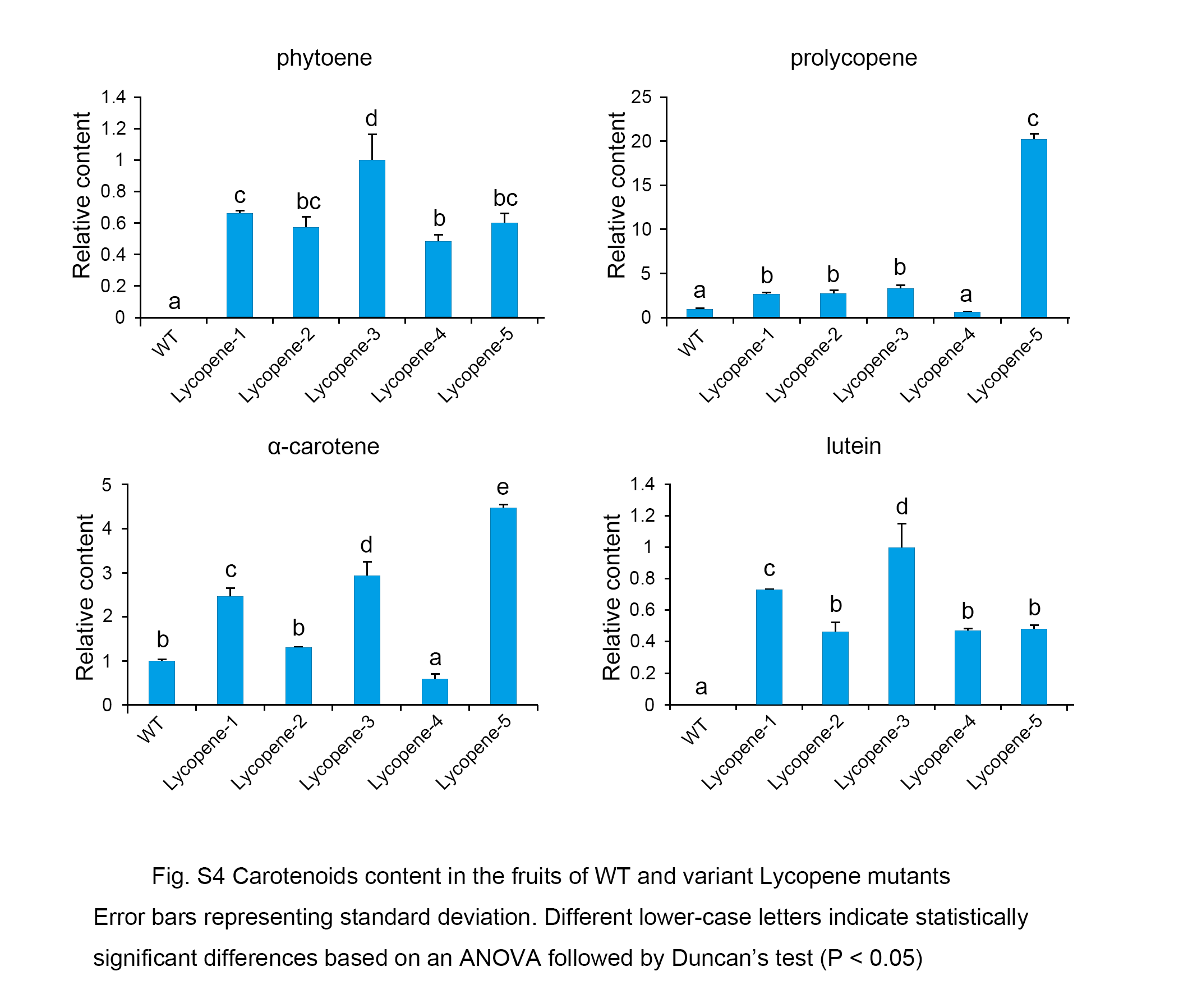

Supplement: Supplementary file 5 [file Image_4.TIF]

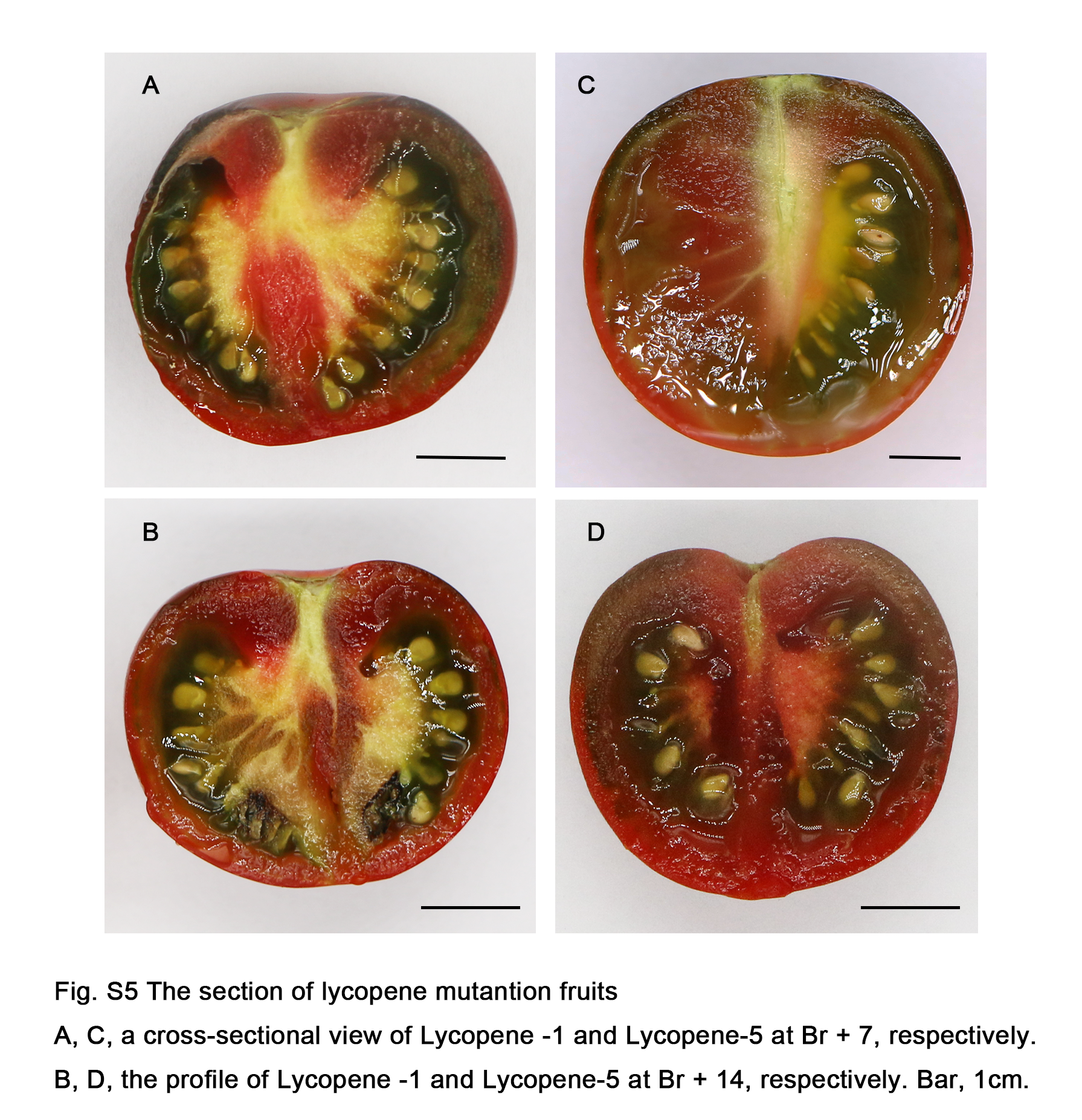

Supplement: Supplementary file 6 [file Image_5.tif]
